# Aquafaba from Korean Soybean I: A Functional Vegan Food Additive

**DOI:** 10.3390/foods10102433

**Published:** 2021-10-13

**Authors:** Youn Young Shim, Yue He, Ji Hye Kim, Jae Youl Cho, Venkatesh Meda, Wan Soo Hong, Weon-Sun Shin, Sang Jin Kang, Martin J. T. Reaney

**Affiliations:** 1 Department of Plant Sciences, University of Saskatchewan, Saskatoon, SK S7N 5A8, Canada; younyoung.shim@usask.ca; 2 Prairie Tide Diversified Inc., Saskatoon, SK S7J 0R1, Canada; 3 Department of Integrative Biotechnology, Biomedical Institute for Convergence at SKKU (BICS), Sungkyunkwan University, Suwon 16419, Korea; kjhkjhmlml@skku.edu (J.H.K.); jaecho67@gmail.com (J.Y.C.); 4 Department of Chemical and Biological Engineering, University of Saskatchewan, Saskatoon, SK S7N 5A9, Canada; yuh885@mail.usask.ca (Y.H.); venkatesh.meda@usask.ca (V.M.); 5 Department of Foodservice Management and Nutrition, Sangmyung University, Seoul 51767, Korea; wshong@smu.ac.kr; 6 Department of Food and Nutrition, College of Human Ecology, Hanyang University, Seoul 04763, Korea; hime@hanyang.ac.kr; 7 A-Life Corp., Goyang 10583, Korea; sangjin321@a-life.co.kr

**Keywords:** aquafaba, soybean, chickpea, emulsifiers, egg replacer, vegan, legume

## Abstract

The substitution of animal-based foods (meat, eggs, and milk) with plant-based products can increase the global food supply. Recently, pulse cooking water (*a.k.a.* aquafaba) was described as a cost-effective alternative to the egg in gluten-free, vegan cooking and baking applications. Aquafaba (AQ) forms stable edible foams and emulsions with functional properties that are like those produced by whole egg and egg white. However, the functional ingredients of AQ are usually discarded during food preparation. In this study, Korean-grown soy (*ver.* Backtae, Seoritae, and Jwinunikong) and chickpea were used to produce AQ. Two approaches were compared. In the first, seed was cooked at an elevated pressure without presoaking. In the second, seed was soaked, then, the soaking water was discarded, and soaked seed was cooked at an elevated pressure. Both approaches produced a useful emulsifier, but the latter, with presoaking, produced a superior product. This approach could lead to a process that involves a small number of efficient steps to recover an effective oil emulsifier, produces no waste, and is cost-effective. The AQ product from Backtae (yellow soybean) produced emulsions with better properties (90%) than AQ produced from other cultivars and produced more stable food oil emulsions. This study will potentially lead to gluten-free, vegan products for vegetarians and consumers with animal protein allergies. This is the first report of the efficient production of AQ, an egg white substitute derived from cooked soybean of known cultivars.

## 1. Introduction

Based on consumer concerns related to health, the economy, food safety, ethics, religion, and environmental issues, there is a trend of reduced consumption of meat and other animal products [1,2]. Conversely, plant-based diets are gaining popularity, and the number of vegetarians and vegans is increasing. The global meat substitute market utilizes several plant-based protein sources which include soy-based, grain-based, vegetable/plant-based, and single-cell protein-based ingredients [3]. Asia is the second-largest market for meat substitutes after North America, where consumption is growing at an annual rate of 4.2% [3]. Due to the growing Asian population and familiarity with soybean foods, soy-based ingredients are emerging as major components of meat alternatives, especially among young people. Similarly, the plant-based protein and hydrocolloid egg substitute market is expanding rapidly and is projected to have a compound annual growth rate of 5.8% for these ingredients over the next 8 years, with revenue expected to reach USD 1.5 billion in 2026 [4]. More and more consumers are opting for vegan protein sources and allergen-reduced plant-based dietary ingredients. Moreover, the demand for high-protein, high-fiber, and low-saturated-fat ingredients such as legume seed products, especially chickpea, lentil, and soybean, is steadily increasing [5].

Soybean, which has been widely used as an ingredient in meat substitutes, contains not only high-quality protein but is also rich in oleic and linoleic acid, which can mitigate the effects of vascular diseases [6]. Despite these nutritional benefits, soybean in the Asian diet is limited to tofu, soymilk, soy sauce and miso. Therefore, it is beneficial to develop and encourage the consumption of products that utilize the excellent components of soybeans in more diverse ways.

AQ is a thick, semi-transparent liquid produced when pulse seed is boiled in water. This byproduct liquid is usually separated from the cooked seed and then discarded. The cooked seed is consumed. AQ contains polysaccharide, protein, and small molecules, which produce a solution that has foamability, emulsibility, gelation, and thickening properties. Recent studies have shown its potential as a vegan, gluten- and cholesterol-free rheological additive in different food recipes such as vegan mayonnaise, meringues, mousse, whipped cream substitute, and sponge cake [7]. Replacing egg and other animal ingredients with AQ provides significant utility and more food options to the vegan community and people who have egg allergies. Exploiting this ingredient also helps to meet the worldwide effort to control global warming and environmental degradation, maintain biodiversity, reduce anthropogenic greenhouse gas emissions, and realize the sustainable utilization of natural resources [8,9,10,11,12,13]. The first peer-reviewed manuscript describing AQ was published in 2018 [14]. Since then, researchers in several groups have described AQ as a novel plant-based rheological additive for food applications [15].

Chickpea AQ properties arise from compounds that leach from chickpea seeds during cooking [7]. Unlike chickpea of pulse, soybean is classified as an oilseed legume rather than a pulse due to its high oil content (about 20%) [16]. Hence AQ from soybean might differ from chickpea AQ due to its different seed and cell wall compounds and structures. AQ produced from soybean might not have the same functionality as that from chickpea. In addition, research related to the AQ composition, functionality, and potential use to improve vegan food quality possible with AQ from specific soybean cultivars remains limited.

In this study, we sought to develop soybean AQ with three types of functional high-protein Korean soybeans and two cooking conditions (with/without presoaking). AQ emulsion and foaming properties were measured to evaluate any influence of the presoaking process on the AQ functional properties and for comparison with chickpea AQ. In addition, the soybean chemical composition and AQ color were measured, as well as the variations of soybean and chickpea AQ.

## 2. Materials and Methods

### 2.1. Materials and Chemicals

**Figure 1:**
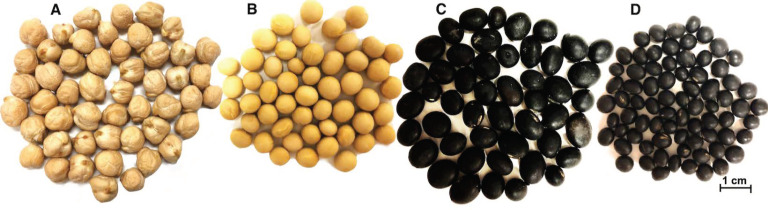
Images of the clean dried seeds used in the AQ production obtained (10 magnification) with a Canon Eos 300D digital camera mounted on a Zeiss Stemi SV 11 light microscope. The images were subsequently processed in Adobe Photoshop 2021 v22.4.3. (**A**) CDC Leader, (**B**) Backtae, (**C**) Seoritae, (**D**) Jwinunikong.

Soybean AQ was produced from three Korean soybean cultivars (*Glycine max* (L.) Merr., *ver*. Backtae and Seoritae; *Rhynchosia nulubilis*, *ver*. Jwinunikong) (Figure 1). A Kabuli type of chickpea (*Cicer arietinum* (L.), *ver.* CDC Leader) was used to produce a standard AQ for comparison with previous studies [17]. Seed of CDC Leader chickpea was generously provided by Dr. Bunyamin Ta’ran from the University of Saskatchewan, Crop Development Centre (CDC, Saskatoon, SK, Canada). All Korean soybean samples were obtained from a local grocer, Nonghyup Hanaro Market (Seoul, Korea). All seeds were stored at room temperature (22 °C) until analyzed. Canola oil (purity 100%; ACH Food Companies, Inc., Terrace, IL, USA) and baking soda (NaHCO_3_; ARM & HAMMER by Church & Dwight Co., Inc., Mississauga, ON, Canada) were purchased from a local supermarket (Walmart, Saskatoon, SK, Canada). Sodium dodecyl sulfate (SDS) was purchased from GE Healthcare (Mississauga, ON, Canada). Anhydrous ether was obtained from Fisher Scientific Co. (Ottawa, ON, Canada). Sodium hydroxide (NaOH) and sodium chloride (NaCl) were purchased from Sigma-Aldrich Canada Ltd. (Oakville, ON, Canada). Concentrated sulphuric acid (H_2_SO_4_, ≥96%, *w*/*w*) and methanol were acquired from EMD Millipore Corporation (Burlington, MA, USA).

### 2.2. Fresh AQ Preparation

The AQ samples were prepared by methods modified from He et al. (2021) [17] and Serventi et al. (2018) [18]. A total of 8 samples (6 soybean AQ samples, 1–3, S1–S3, Table 1) were produced in this study to evaluate the effects of a soaking treatment on AQ functionality. Subsamples of AQ were frozen to –20 °C, then dried in a freeze dryer (FreeZone 12 L Console Freeze Dryer with Stoppering Tray Dryer, Labconco Corporation, Kansas City, MO, USA) until the sample temperature rose to –5 °C, indicating that the sample had been thoroughly dried. The freeze-dried AQ yield was measured.

#### 2.2.1. AQ Produced without Presoaking

In total, 200 g of each seed were rinsed with distilled water and then mixed with 300 mL distilled water (1:1.5, *w*/*w*) in 500 mL sealed glass jars and cooked in a pressure cooker (Instant Pot^®^ 7 in 1 multi-use programmable pressure cooker, IP-DUO60 V2, 6 quart/L) at 115–118 °C (an autogenic pressure range of 70–80 kPa) 90 min. Subsequently, the jars of cooked soybean and chickpea were cooled by holding at room temperature for 24 h. Cooled AQ was drained from the cooked seed using a stainless-steel mesh kitchen strainer and stored in a freezer (–20 °C). Each procedure was replicated three times.

#### 2.2.2. AQ Produced with Presoaking

In total, 200 g of each seed were washed with distilled water and pre-soaked at a ratio of 1:3 (*w*/*w*, chickpea) and 1:4 (*w*/*w*, soybean) at 4 °C for 16 h. Subsequently, the presoaked seed was rinsed with distilled water and mixed with the same amount of distilled water (1:1, *w*/*w*) in sealed glass canning jars. The pressure cooker parameters were the same as described in Section 2.2.1, except for chickpea AQ, which was cooked for 30 min, the optimum cooking time for this AQ reported by He et al. (2021) [17].

### 2.3. Chemical Properties

The moisture contents of whole soybean seed samples were measured by the ASAE S352.2 air oven drying method (103 °C, 72 h, 15 g) [19]. Whole seed was ground with a disc mill prior to proximate composition analysis. Analyses of the crude protein, crude fat, ash, and crude fiber were performed using Association of Official Analytical Chemists (AOAC) methods [20]. In brief, the nitrogen content was analyzed by combustion AOAC Method 990.03 [20] using a LECO (Saint Joseph, MI, USA) nitrogen analyzer. Protein content was calculated as the nitrogen content (N) multiplied by a conversion factor of 6.25. Fat was extracted from ground samples according to AOAC method 920.39 [20] using anhydrous ether in a Soxhlet apparatus (Extraction system B-811, BÜCHI Labortechnik AG., Switzerland). Ground seed samples were weighed (2 g) onto filter paper, which was then placed in a cellulose Soxhlet extraction thimble and washed five times with 20 mL distilled H_2_O each time. After drying in an oven at 102 °C for 2 h, oil was extracted over 5 h in a Soxhlet apparatus with anhydrous ether. Seed ash content was determined by AOAC method 942.05 [20]. Samples were weighed (2 g) in separate pre-weighed porcelain crucibles and placed in a preheated furnace (600 °C) for 2 h. The crucibles were then transferred to a desiccator, cooled, and reweighed. The sample weight remaining after ignition of a 2 g sample was regarded as ash content. The crude fiber content was determined by AOAC method 962.09 with minor modifications [20]. The samples were digested with 1.25% (*w*/*v*) boiling H_2_SO_4_ (30 min), followed by 1.25% (*w*/*v*) boiling NaOH (30 min), and washed with boiling water and methanol. The samples were then dried to a constant weight and the residue burned. The weight loss on ignition of the dried residue was regarded as the crude fiber content. The carbohydrate content was determined by subtracting the total percentage of protein, fat, fiber, and ash components from 100%.

### 2.4. AQ Foaming Properties

The foaming capacity and foaming stability of the freshly prepared liquid AQ were determined according to the methods described by Martinez et al. (2016) [21] and Mustafa et al. (2018) [22]. AQ (5 mL) was diluted with 10 mL water in a 150 mL graduated measuring cup, then whipped for 2 min at speed setting 10 using a Kitchen Aid Ultra Power Mixer (Kitchen Aid, St. Joseph’s, MI, USA) with a 4.3 L stationary bowl. The foam volumes of whipped samples were measured at time 0 (*V*_F0_) and after 30 min (*V*_F30_), and the foaming capacity and foaming stability were calculated by Equations (1) and (2), respectively.

(1) $$\%\mathrm{Foaming}\;\mathrm{capacity}\;=\frac{V_{F0}}{V_{\mathrm{sample}}}\times 100$$

(2) $$\%\mathrm{Foaming}\;\mathrm{stability}\;=\frac{V_{F30}}{V_{F0}}\times 100$$

### 2.5. AQ Emulsion Properties

#### 2.5.1. Oil Emulsion Preparation

Canola oil (14 g) was added dropwise to the freshly prepared liquid AQ (6 g) and simultaneously mixed using a Kitchen Aid Ultra Power Hand Mixer (Kitchen Aid, St. Joseph’s, MI, USA) for 2 min to obtain a food oil emulsion with 70% oil content.

#### 2.5.2. Emulsion Capacity

Each AQ oil emulsion was diluted 100-fold with 0.1% SDS (*w*/*v*), and an UV-Vis spectrophotometer was used to determine transmittance at 500 nm immediately after dilution. The subsequent steps and the calculation of the emulsion capacity values were in accordance with He et al. (2019) [23].

#### 2.5.3. Emulsion Stability

Emulsions (*F*_0_ = 10 g) were transferred to sealed 15 mL centrifuge tubes and then centrifuged at 1860× *g* for 15 min. The weight of the emulsified fractions (upper layer, *F*_1_) was measured after centrifugation [17]. The emulsion stability (%) at room temperature was calculated by Equation (3) [24].

(3) $$\%\mathrm{Emulsion}\;\mathrm{stability}\;=\frac{F_{1}}{F_{0}}\times 100$$

### 2.6. Color

The color of the AQ samples was determined using a Hunter lab ColorFlex spectrophotometer (Hunter Associates Laboratory, Inc., Reston, VA, USA). The instrument was standardized with black and white tiles (X = 79.1, Y = 83.8, Z = 89.4), and a green tile (*L* = 52.9, *a* = –5.8, *b* = 12.9) as a color check. The AQ color was represented by L* (lightness/darkness), a* (redness/greenness), and b* (yellowness/blueness).

### 2.7. Statistical Analysis

The experiments were conducted in triplicate, and the data were presented as mean ± standard deviation (SD, *n* = 3). The analytical results were processed with Microsoft Excel^®^ 2018. Statistical analyses were implemented through the Statistical Package for the Social Science (SPSS) version 25.0 (IBM Corp., Armonk, NY, USA). The analysis of variance (ANOVA) and Tukey’s post hoc tests were used to evaluate the significant differences of chickpea/soybean seed composition, AQ freeze-dried sample yield, freshly prepared AQ moisture content, functional properties, and color. Statistical significance was accepted at *p* < 0.05.

## 3. Results and Discussion

### 3.1. Chemical Characteristics of Dried Seeds

During cooking, the seed coat of soybean acts as a selective membrane that controls the mass transfer, affecting the AQ composition and, consequently, cooking water (AQ) functional properties [25]. The physical properties of the seed coat depend on the genotype and environmental conditions (temperature, soil, and moisture) during seed maturation and storage [26]. The main chemical components of different chickpea/soybean cultivars are shown in Table 2. Seoritae had the highest seed moisture content (10.52 ± 0.07%) among seed of the three soybean cultivars. The seed carbohydrate content and crude fiber content of the three soybean cultivars were not significantly different. Protein was the main component of all soybean cultivars. Seoritae and Jwinunikong had the highest crude protein content (44.8 ± 0.2 g/100 g and 44.4 ± 0.2 g/100 g seed, respectively, *p* < 0.05), followed by Backtae (40.6 ± 0.3 g/100 g seed), which had the highest fat and ash content (18.4 ± 0.7 g/100 g seed and 4.7 ± 0.3 g/100 g seed, respectively). The carbohydrate content of seed from the chickpea cultivar (CDC Leader) was more than double that of the seed from the three soybean cultivars. The protein and fat contents of the chickpea seed were less than half those of soybean seed (*p* < 0.05, Table 2). These observations agree with the previous study by Sharma et al. (2014) [27], who measured the chemical compositions of seeds from eight different soybean genotypes. In their study, the genotypes contained crude protein and oil in the range of 39.4–44.4% and 14.0–18.7%, respectively. In contrast, lower protein content (31.4–37.0%) and higher fat content (17.9–21.9%) were observed in seeds of 10 soybean cultivars [28]. Nevertheless, the ash content (4.86–5.49%) and fiber content (7.03–8.78%) agree with our results.

### 3.2. AQ Produced from Different Seed and Conditions

**Figure 2:**
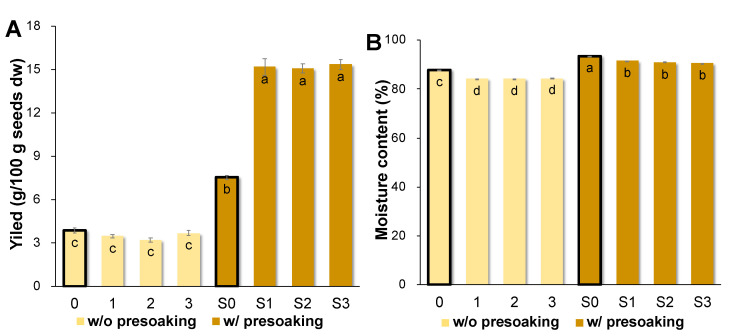
(**A**) The yield of the freeze-dried AQ (g/100 g seed, dw) and (**B**) moisture content (%, ww) of the fresh AQ prepared from different soybean cultivars and cooking conditions. Bold bar graphs are each group of controls (0 and S0), respectively. Values followed by different letters (a–d) within a property are significantly different (*p* < 0.05) according to Tukey’s test.

The moisture content of fresh AQ is over 90% [23]. It is highly desirable to concentrate or dry AQ to reduce shipping costs and the need for storage space. More importantly, AQ is potentially used as a food ingredient, so food safety is the primary concern. AQ is rich in carbohydrates and protein, which are nutrients for microorganisms [29]. Therefore, a drying process such as freeze-drying would be desirable to reduce the AQ water activity, maintain its quality, and increase its shelf-life. The AQ prepared from three Korean soybean cultivars with the presoaking process (S1–S3) produced significantly higher yields than other freeze-dried AQ products (*p* < 0.05, Figure 2A). In the presoaked group (S0–S3), the freeze-dried AQ yield ranged from 8.0 g/100 g dw seed for chickpea to 16.2 g/100 g dw seed, with the highest yield produced by S1–S3 (*p* < 0.05) (Figure 2A). In the group without presoaking (0–3, Figure 2A), the freeze-dried AQ yields (3.38–4.11 g/100 g seed) were not significantly different among the different chickpea and soybean cultivars. The unsoaked seed absorbed more water during cooking, leading to decreased AQ yield.

Total moisture content (%, wet weight, ww) of AQ produced from the presoaked seed (S0–S3, Figure 2B) was significantly higher than that of AQ from seed without presoaking (0–3, Figure 2B). The chickpea AQ in both groups (S0 and 0) had higher moisture content than soybean AQ samples (S1–S3, 1–3, *p* < 0.05). The AQ moisture content was not affected by the soybean cultivar (*p* < 0.05). High yields of AQ with low moisture content (high dry matter content) could be of greater commercial value.

Considering the unique physicochemical characteristics and immersion conditions of chickpea and soybean seed, it was unreasonable to produce soybean AQ under conditions optimized to produce chickpea AQ. According to the method described by Serventi et al. (2018) soybean AQ was produced by boiling seed for 90 min [18]. This cooking time was chosen in the current study for AQ produced both with and without presoaking treatment (S1–S3, 1–3). This helps in making comparisons of current results with similar conditions described in previous studies [15,18,30,31]. The optimum cooking time to maximize chickpea AQ emulsion properties was 30 min according to He et al. (2021) [17]. In our first experiment design, we cooked the chickpea for 30 min without presoaking, which is the optimum cooking time stated by He et al. (2021) [17]. However, we found that without presoaking, the 30 min cooking time was not sufficient to soften the seed. Furthermore, foaming and emulsion properties of AQ produced this way were significantly diminished (data not shown). Therefore, to achieve AQ with good function, unsoaked chickpeas were cooked for 90 min (0), and soybeans were cooked for 90 min (1–3). For the presoaked chickpeas and soybeans, the cooking times were 30 min (S0) and 90 min (S1–S3), respectively.

### 3.3. Color Analysis

**Figure 3:**
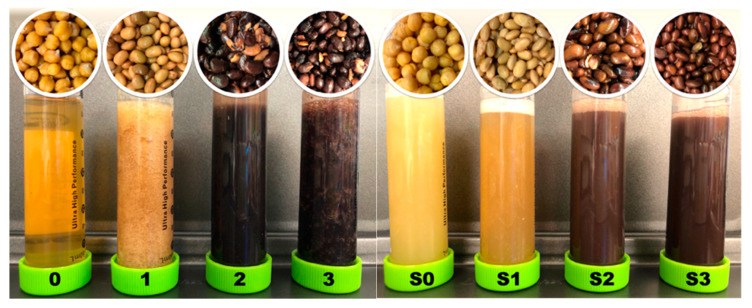
AQ samples separated from chickpea and soybeans. L–R, seed images: seed remaining after cooking and separation of the AQ, w/o presoaking AQ samples (0–3) and w/presoaking AQ samples (S0–S3); L–R, tube images, w/o presoaking AQ samples (0–3) and w/presoaking AQ samples (S0–S3).

Color and turbidity of AQ varied depending on the type of seed and treatment (Figure 3). In the present study, dried AQ produced from the Kabuli chickpea cultivar (*C. arietinum* L.), CDC Leader, and from yellow soybean (*G. max* (L.) Merr., Backtae) were creamy yellow. Whereas the AQ 0 prepared from CDC Leader without presoaking was almost transparent (L* = 46.35 ± 0.06, a* = 2.55 ± 0.01, b* = 21.47 ± 0.06, *p* < 0.05), the AQ samples prepared from large and small black soybeans Seoritae (*G. max* (L.) Merr., AQs 2 and S2) and Jwinunikong (*R. nulubilis*, AQs 3 and S3), respectively, were dark brown to black with high turbidity. With larger L* and a* values, S2 and S3 were significantly brighter and redder compared with AQs 2 and 3, indicating that the presoaking process might remove some dark brown seed pigments. This dark brown color may be caused by anthocyanins in the large and small black soybeans seed coats, which might migrate into the AQ product during cooking [32].

The densities of the AQ from the pre-soaked untreated (0–3) and the pre-soaked group (S0–S3) were 1.053–1.065 and 1.015–1.036, respectively, and there was a significant difference between the two groups (*p* < 0.05, data not shown). The densities of the AQ from commercially canned chickpea ranged from 1.009 to 1.180 g/mL [14].

Various colors were observed in the AQ after each AQ cooking process (Table 3). L* values indicate a significant decrease (*p* < 0.05) in both AQ production cooking processes with dried seed. In particular, the darkest L* was observed in the AQ from unsoaked black soybeans (2, 3). Since AQ contains both proteins and carbohydrates, the blackening phenomenon may be related to the Maillard reaction between these components or could arise from extracted anthocyanin.

Increased L* values for these samples during storage indicate that both darkened as storage increased. The parameter a* also changed during storage, and the decrease in the overall a* value after cooking yellow legumes is thought to be related to chlorophyll [33]. Two groups of yellow seed (CDC Leader and Backtae) and black seed (Seoritae and Jwinunikong) showed different results in parameter b*. After the production of AQ, the yellowness of each group of yellow legumes decreased, but the blueness of black soybeans before cooking gradually increased after cooking. According to Wang, soybean contains high amounts of isoflavones, and these are likely to be responsible for the high b* values [34]. It has been reported that the availability of soybean isoflavone glycosides and aglycones can be increased during cooking and processing [34]. In addition, during the heating process, the distribution of isoflavones in soy products changes, thereby increasing absorption in the body [35]. It was reported that β-glucosidase increased the amount of free isoflavone in soybean during soaking in water [36]. Therefore, the b* value of the black soybean AQ produced with soaking (S2, S3) was higher than that of the AQ produced without presoaking (2, 3).

### 3.4. AQ Foaming Properties

**Figure 4:**
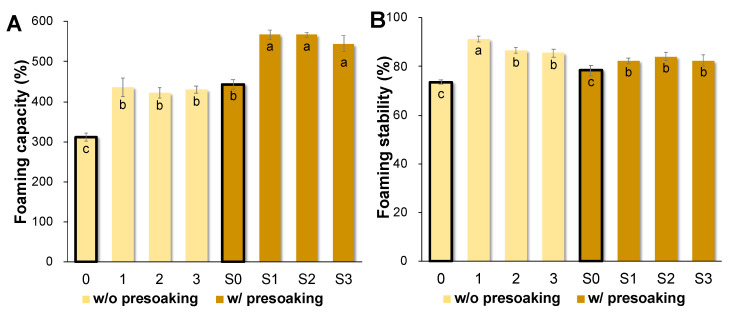
Foaming properties of the AQ produced from chickpea and three Korean soybean cultivars. Bold bar graphs are each group of controls (0 and S0), respectively. Each foaming property value marked with a different letter (a–c) is significantly different (*p* < 0.05) from the others marked with a different letter according to Tukey’s test. (**A**) Foaming capacity and (**B**) foaming stability.

The AQ samples produced with a presoaking treatment (S0–S3) had significantly greater foaming capacity than those prepared without presoaking (0–3) (*p* < 0.05, Figure 4A). In particular, the foaming capacities of all soybean AQ preparations were similar but greater than chickpea AQ (544 ± 19% to 567 ± 6% vs. 443 ± 14% in presoaking group, 422 ± 14% to 437 ± 23% vs. 311 ± 10% unsoaked seed; *p* < 0.05, Figure 4A). However, except for the AQ made from Backtae without presoaking (0), which had the highest foaming stability (91.34 ± 1.23%; *p* < 0.05, Figure 4B), the AQ foaming stability was not significantly influenced by the cooking conditions with/without a presoaking treatment. In all soybean AQ samples, the foaming stability was generally more than 80% (82.35 ± 1.1% to 91.34 ± 1.2%; Figure 4B). The foaming capacity of the Backtae AQ produced by presoaking (S1, 567 ± 12%) in the current study was superior to that reported by Serventi et al. (2018) [18] (165 ± 2%, when calculated by the equation in the current study). In their study, yellow soybean was cooked in boiling water rather than in a pressure cooker at temperatures greater than 100 °C. Extracting the AQ at an elevated temperature and pressure might change the composition to further influence its foaming properties [7].

The soybean AQ foaming capacity was superior to that of chickpea AQ (297%), commercial egg white liquid (281%), and fresh egg white (311%) [22]. Due to its preferred foaming properties, chickpea AQ has been successfully utilized in vegan sponge cake, French meringue, and mousse [15,22,31,37,38,39]. Therefore, with the observation that soybean protein has higher foam expansion compared with other legume proteins [40,41], AQ produced from three types of Korean soybeans are expected to be utilized as a plant-based foaming agent in the development of new food formulations with added flavor and nutrition to counter the nutritional imbalance of vegan food.

### 3.5. AQ Emulsion Properties

**Figure 5:**
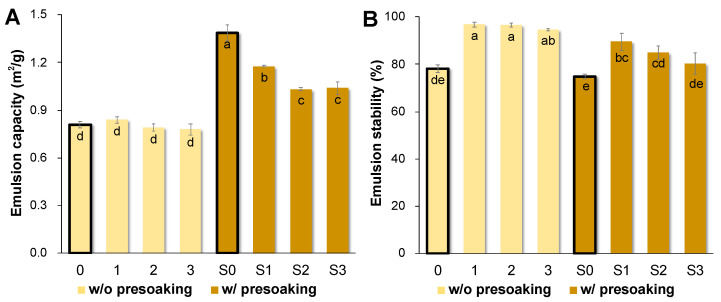
(**A**) Emulsion capacity and (**B**) stability of the AQ prepared from different seed types. Bold bar graphs are each group of controls (0 and S0), respectively. All emulsions prepared in this study dispersed easily in water and were thereby confirmed to be oil/water emulsions. Each value marked with a different letter (a–e) is significantly different (*p* < 0.05) from the others marked with a different letter according to Tukey’s test.

The emulsion capacities of the AQ samples (S0–S3) produced with a presoaking treatment were significantly greater than those achieved without presoaking (0–3) (*p* < 0.05, Figure 5A). In the presoaking group, chickpea AQ had the highest emulsion capacity (S0, 1.39 ± 0.05 m^2^/g), followed by Backtae AQ (S1, 1.18 ± 0.004 m^2^/g), Jwinunikong AQ (S3, 1.04 ± 0.05 m^2^/g), and Seoritae AQ (S2, 1.03 ± 0.01 m^2^/g) (*p* < 0.05, Figure 5A). No significant differences were observed between the chickpea and soybean AQs produced without the presoaking steps (0–3, 0.78 ± 0.03 m^2^/g to 0.84 ± 0.02 m^2^/g; *p* < 0.05, Figure 5A). Interestingly, all Korean soybean AQ samples produced without a presoaking step (1–3) achieved higher emulsion stability than those produced with a presoak treatment (94.66 ± 0.46% to 96.80 ± 1.06% vs. 80.38 ± 4.39% to 89.49 ± 3.72%; *p* < 0.05, Figure 5B). In addition, soybean AQ samples (S1–S3, 1–3) in both groups showed similar or relatively higher emulsion stability than chickpea AQ (S0, 74.91 ± 0.94%; 0, 78.05 ± 1.66%; *p* < 0.05). He et al. (2018) determined that AQ emulsion stability was inversely correlated to AQ moisture content, which might explain results of the current study. Essentially, AQ samples 1, 2, and 3 with lower moisture content had higher emulsion stability than samples S1, S2, and S3, respectively. However, chickpea AQ prepared by both methods had different moisture contents, but their emulsion stability showed no significant differences (*p* < 0.05). Since samples 0 and S0 were produced with different cooking times (90 min vs. 30 min), prolonged cooking time might induce protein denaturation and starch swelling/gelatinization, as well as increase the cell wall breakdown and transfer of compounds to the AQ [7,42].

The results of the current study contrast with previous research, in which yellow soybean (Backtae) AQ had a higher emulsion capacity of 20.3 ± 1.8 m^2^/g and lower emulsion stability of 49.3 ± 3.7% [18]. In a previous study, the emulsion capacity was measured on emulsions prepared by homogenizing 20 mL of soybean AQ and 20 mL of canola oil for 1 min. To measure the emulsion stability, 2.5 g freeze-dried soybean AQ was homogenized with water and canola oil for 1 min, whereas in the current study, AQ and canola oil (6:14, *w*/*w*) were mixed by a hand mixer for 2 min. Several factors, including the oil and emulsifying agent concentrations, homogenization process, and prolonged mixing time, might play important roles in influencing AQ emulsion stability [43,44].

When comparing the current study with others that use similar cooking methods, freeze-dried chickpea AQ has been applied as a plant-based emulsifier in vegan mayonnaise analog with comparable pH, color, and stability to traditional egg yolk mayonnaise [17]. In addition, Lafarga et al. (2019) [39] developed a chickpea AQ vegan mayonnaise with high flavor and texture scores. Moreover, the AQ-to-oil ratio was optimized (15/80%) to obtain a stable emulsion with comparable droplet size distribution to the microstructure of real mayonnaise [45]. Therefore, we predict that soybean AQ can also be applied as a plant-based emulsifier to produce vegan mayonnaise analogs. In addition, yellow soybean (Backtae) is one of the most traded agricultural products worldwide due to its high productivity and profitability [46]. It has been widely used in the production of tofu, soy milk, and soy sauce. The Backtae AQ by-product available from manufacturing processes using this seed is large. Thus, Backtae AQ might be the most widely recycled soybean AQ source. This is very important in securing the food sources recycling and preventing environmental pollution.

## 4. Conclusions

In this study, AQ was prepared from three Korean soybeans and two cooking conditions (with/without presoaking, sample S0–S3, 0–3). The AQ samples produced with a presoaking treatment had a greater yield after freeze-drying when compared with the AQ samples prepared without presoaking. The yield was coupled with mostly superior functional performance. These findings reveal that presoaking plays a significant role in contributing to AQ functional properties. The freeze-dried soybean AQ yield was more than double that of the chickpea AQ. In addition, although the soybean seed chemical composition had significant differences with the chickpea seed, the soybean and chickpea AQ samples had similar foaming and emulsifying properties. Therefore, like chickpea AQ, which has been studied previously, soybean AQ can be regarded as a potential plant-based emulsifier and foaming agent. Further studies related to Korean soybean AQ will focus on AQ standardization, the determination of AQ functional components, physicochemical properties, and applications of AQ in real food products such as vegan mayonnaise analogs and sponge cakes. Based on these research results, it will be possible to respond to the dietary needs of vegan food and egg allergies using a soy-based alternative to egg or other animal sources as a food rheological additive.

## Figures and Tables

**Table 1:** AQ preparation conditions.

	Species/Cultivar (Code)	Canadian Chickpea ^1^	Korean Soybeans ^2^		
		*Cicer arietinum* (L.)	*Glycine max* (L.) Merr.		*Rhynchosia nulubilis*
Group		CDC Leader	Backtae	Seoritae	Jwinunikong
w/o Presoaking AQ ^3^		0	1	2	3
w/Presoaking AQ ^4^		S0	S1	S2	S3

^1^ Canadian chickpea as a control (light gray column): CDC Leader; ^2^ Korean soybeans: (1) Backtae: yellow soybean, (2) Seoritae: large black soybean, (3) Jwinunikong: small black soybean; ^3^ w/o: without; ^4^ w/: with.

**Table 2:** Characteristics of the dried seeds.

Characteristics	Unit	CDC Leader ^1^	Backtae	Seoritae	Jwinunikong
Moisture	%	8.86 ± 0.07 ^c^	9.30 ± 0.02 ^b^	10.52 ± 0.07 ^a^	9.26 ± 0.11 ^b^
Carbohydrate ^2^	g (100 g/dw)	65.4 ± 2.01 ^a^	29.14 ± 0.78 ^b^	29.16 ± 0.32 ^b^	27.88 ± 1.32 ^b^
Crude protein ^3^	g (100 g/dw)	20.9 ± 0.09 ^c^	40.59 ± 0.28 ^b^	44.77 ± 0.15 ^a^	44.43 ± 0.22 ^a^
Fat ^4^	g (100 g/dw)	6.49 ± 0.47 ^c^	18.40 ± 0.69 ^a^	14.91 ± 0.30 ^b^	14.51 ± 0.37 ^b^
Ash ^5^	g (100 g/dw)	3.01 ± 0.08 ^d^	4.65 ± 0.26 ^a^	4.17 ± 0.14 ^c^	4.52 ± 0.01 ^b^
Crude fiber ^6^	g (100 g/dw)	4.32 ± 0.26 ^b^	7.27 ± 0.47 ^a^	6.99 ± 0.32 ^a^	8.66 ± 1.43 ^a^

Values (mean ± SD) within rows followed by the same letter do not statistically differ at *p* < 0.05 by Tukey’s test. ^1^ CDC Leader’s data were modified from He et al. (2019) [23] as a control (light gray column); ^2^ Carbohydrate content was obtained by subtracting the sum of crude protein, fat, ash, and crude fiber from 100; ^3^ The factor N × 6.25 was used to convert nitrogen into crude protein; ^4^ AOAC method 920.39; ^5^ AOAC method 942.05; ^6^ AOAC method 962.09.

**Table 3:** Color parameters of all samples used for AQ production.

Group	L*	a*	b*
*Seed*			
CDC Leader	62.98 ± 0.19 ^a^	8.54 ± 0.02 ^a^	25.77 ± 0.10 ^a^
Backtae	66.30 ± 0.19 ^a^	6.77 ± 0.10 ^b^	24.17 ± 0.12 ^a^
Seoritae	34.53 ± 0.11 ^b^	–0.64 ± 0.08 ^g^	–4.42 ± 0.06 ^d^
Jwinunikong	34.76 ± 0.10 ^b^	–0.61 ± 0.06 ^g^	–4.89 ± 0.07 ^d^
*w/o Presoaking AQ*			
0	46.35 ± 0.06 ^b^	2.55 ± 0.01 ^d^	21.47 ± 0.06 ^a^
1	33.08 ± 0.22 ^b^	5.27 ± 0.17 ^c^	14.43 ± 0.14 ^b^
2	16.10 ± 0.04 ^d^	2.05 ± 0.24 ^e^	–1.75 ± 0.10 ^c^
3	17.08 ± 0.15 ^d^	2.20 ± 0.03 ^e^	–1.26 ± 0.12 ^c^
*w/Presoaking AQ*			
S0	39.30 ± 0.14 ^b^	0.79 ± 0.02 ^c^	15.55 ± 0.40 ^b^
S1	38.99 ± 0.24 ^b^	1.25 ± 0.17 ^f^	11.33 ± 0.06 ^b^
S2	27.61 ± 0.40 ^c^	4.76 ± 0.12 ^d^	1.00 ± 0.18 ^c^
S3	26.19 ± 0.54 ^c^	5.12 ± 0.25 ^c^	1.08 ± 0.07 ^c^

^a–g^ Values followed by different letters within a column are significantly different (*p* < 0.05) according to Tukey’s test. L*, brightness/darkness; a*, (+) redness/(–) greenness; and b*, (+) yellowness/(–) blueness.

## Data Availability

The data of the current study are available from the corresponding author on reasonable request.
